# Case Report on the Use of the Waldon Approach on an Adult with Severe to Moderate Intellectual Disability with Autistic Tendencies

**DOI:** 10.3389/fpubh.2016.00050

**Published:** 2016-03-29

**Authors:** Walter G. Solomon, Eran Greenbaum

**Affiliations:** ^1^Feuerstein Institute, Jerusalem, Israel; ^2^Princess Basma Centre for Children with Disabilities, Jerusalem, Israel; ^3^Maon Roglit, Neve Michael, Israel

**Keywords:** Waldon Approach, autism spectrum disorder, autism therapy, intellectual disability, cognition, social behavior

## Abstract

This clinical case report describes a patient diagnosed with severe to moderate intellectual disability with autistic tendencies, resident in a home for adults with a range of disabilities. She had been resident for 18 years prior to intervention by the author when she was 48 years of age. The author worked with her from June 25, 2013 until January 12, 2015 for a total of 55 Waldon Approach (1), movement-based lessons each of about 45 min of which 33 were documented by video. This report describes changes in her cognition and her social behavior at a time when there were no other changes in her life. As far as the author is aware, this is the first clinical case report on the Waldon Approach to appear in a peer-reviewed journal and is unique in that most of the work using the approach is with children who are usually receiving other therapies at the same time as their Waldon Lessons, making it harder to evaluate the attribution of change. During the period of this report, she received no other therapy or intervention beyond that provided at Maon Roglit, which itself had not changed during this period. The patient remains without speech, but there has been real, meaningful, and noticeable change in her life from which she appears to derive pleasure. There has been a significant improvement in the patient’s group participation, facial expression, and general demeanor.

## Introduction

We describe a patient treated with the Waldon Approach, developed by UK neurologist, Dr. Geoffrey Waldon, which is based on the premise that all early learning is derived from movement. Children with cognitive and/or emotional delays are likely to have missed movements, which are natural to typically developing children (TDC). Abnormalities in movement interfere with the cascade of typical development. In the Waldon Lesson, the practitioner aids or causes the patient to make the moments appropriate for his or her developmental age, often very different from the chronological age, in order to aid positive change in his or her cognition and emotional development.

The patient has been a resident of Maon Roglit, a home for adults with a range of disabilities since March 1995, and her diagnosis on entry was severe intellectual disability. She is a member of a group of approximately 12 adults who participate in a range of activities based on gardening but also including other group activities, including music and movement activity, story telling, special activity days, etc. They have a full time manager, the second author of this paper, who directs the activities and helps where necessary with dressing, toileting, and general care. He has a full time assistant. Meals are provided in a communal dining hall.

The second author described the patient’s activities from the time he joined the staff of Maon Roglit in March 2011 up to the time that her Waldon Lessons started, as follows: “The patient always sat on the edge of the group and did not participate in any of the activities. She was silent, had no speech and gave no trouble. It was really hard to communicate with her. She was extremely low in her self-confidence and tended to stay attached to her carer. She barely showed what she wanted and could speak about two or three words, so she was cognitively capable of speech but chose not to speak for unknown reasons.”

“The patient is musically talented and occasionally would sing a whole song, but stopped immediately when paid attention to. She rarely participated in anything, but she does like to thumb through and turn pages of different newspapers. It seemed that she did not look at pictures but liked to feel the paper. The patient tended to catatonic emotions and catatonic-type behavior, and mostly showed a happy and smiling face in different emotional situations, but knew how to show her opinion about different things that she liked or not. She did not participate at all in group activities, did not show interest in the activities and could not sit down for the whole time, but tended to get up and walk around. She liked just a few individual activities like playing ball or going for a walk, and helping put certain things back to their places or move tables and chairs as needed.”

## Background

### Methodology and Treatment

Geoffrey Waldon, a neurologist working in Manchester from the 1970s until his death in 1989, developed a theory of child development based on his close observation of children over many years. He was one of the first to recognize that meaning comes from movement and that children’s early understanding is derived from their early movements *in utero* and from birth onward. As they grow and become heavier and longer limbed, these movements become more effortful as they continue to reach out and explore and slowly learn about their environment.

“Meaning from movement” is an expression Geoffrey Waldon used constantly; it is foundational to his theory of learning. Waldon believed that movement is the most consistent and regular source of experience and provides the structure of understanding, which develops alongside the movements. He wrote: “The present paper [*Meaning from Movement*] (2) is concerned with the forces which bring into being the highly improbable patterns from which the world of the human observer, indeed the identity of the observer himself, is created and which, despite their individual isolation, is nevertheless shared by all humans as the basis of understanding.”

Piaget (3) had previously written in *The Psychology of the Child*: “the sensory-motor structures constitute the source of the later operations of thought. This means that intelligence proceeds from action as a whole, in that it transforms objects and reality, and that knowledge whose formation can be traced in the child, is essentially an active and operatory assimilation.”

Both Waldon and Piaget were saying, over 30 years ago, that it is the movement which creates understanding. From the child in the womb, to the new-born, to the infant, to the toddler, then child, and then the adolescent, typical children move to learn. One researcher, Sperry (4) went so far as to say “The sole product of brain function is motor co-ordination.” And it is not only in early development that movement matters but also many children with developmental delays have continued motor problems – problems in moving their bodies as they wish.

As they grow, TDC will gradually acquire and dispose of a whole variety of objects with different textures, mass, material, and shape. They will bang and scrape any item that comes to hand. All this and more will be done spontaneously, in an increasingly effortful manner without adult intervention or approval.

Atypical children may have missed these critical developmental steps and will consequently have gaps in their understanding. The Waldon Lesson seeks to recreate the conditions encountered by typical infants as their own motivation takes them through the learning processes, and they naturally and without external direction develop and apply the fundamental movements discussed above. For the developmentally delayed children, all of these indispensable sensory–motor steps are introduced in the Waldon Lesson, which becomes the vehicle or scaffold for them to encounter and practise all the development stages, which they have missed.

The above applies equally to adults who have for one reason or another failed to develop their general understanding. So in the author’s work at Maon Roglit, he ignored the chronological age of the patients and worked in a way that is entirely congruent with their developmental age. Through these lessons, even after so many years of relative neglect, lives can be improved, and patients can become more open to increased activity and sociability in their daily activities.

The author started to work with the patient and three other residents of Maon Roglit on June 25, 2013 using the Waldon Approach and giving weekly Waldon Lessons to each resident individually for approximately 45 min per session.

One patient dropped out after 8 weeks, although he was responding well and a replacement patient was discontinued after 10 months as he was generally uncooperative and did not appear to be benefiting from treatment. The next replacement patient only had 3 months of lessons before the author’s contract ended. The author had started working at Maon Roglit as a volunteer, but it is perhaps significant that after 4 months management asked him to accept payment for his services. The contract ended in January 2015, and the second author is now training to become a Waldon practitioner.

The author wrote to the director of Maon Roglit on leaving:

Thank you for giving me the opportunity to work with some of your residents over the past year and a half. It was most interesting for me to see their development, as apart from their daily activities I believe they had no other formal therapy. And they improved recognizably.

Patient 1 amazed his family in greeting them by name

Patient 2 (The patient described in the study) now participates in most of the activities and is noticeably more contented

Patient 3 is just starting an amazing transformation and becomes more and more involved in daily activities

It is early days for Patient 4 but I am sure that this would also be good for his anxieties.

## Discussion

### Assessments and Results

After only 10 lessons, the second author reported that the results showed in the patient’s daily life where she is more included in activities. He confirmed that “after four months she showed more confidence among her peer residents and tended slightly less to follow her carer everywhere.”

He further commented on the enormous progress she had made in a year – now joining in all the activities, for example, spreading sandwiches and dancing at the party. The patient shows more confidence and participation in group activities. She is more open to hold hands during a dance with another resident that is not her carer. The patient expanded slightly her spoken vocabulary and tends to sing more. We feel that she understands us better, but in general, there is still a big difference between her seemingly high-level understanding and low-level activity. The patient seems to be calmer and more active in helping the carer and doing tasks and has more confidence in going by herself to the Waldon Approach lessons or music lessons – the carer, making sure that she arrives by watching her from far away or calling by phone. After working with the patient for approximately 3 years before she started the Waldon Approach lessons, there is certainly visible progress in general and in specific tasks or situations. The last diagnosis committee in December 2014 diagnosed her with moderate to low intellectual disability.

Maon Roglit’s Diagnosis Committee made assessments in January 2006 and in December 2014.

In January 2006, the patient had an evaluation by a social worker, psychologist, MD and psychiatrist with telephone contact with Z (older sister).

The Social Worker reported: the patient looked at a newspaper all the time, No speech or eye connection, Mother died in 1982, Father still alive, when she goes home she sits and turns pages of a phone book for example and does not move around much.

Born 2 months premature, she was hospitalized very young for diarrhea, her mental development was delayed, and as a child, she was aggressive. In 1977, she had a psychological check and experienced regression in talking and made no connection with others. She used to talk a few words and sing a little and she stopped. Her diagnosis was not autism, but organic psychotic development. In 1991, she was described with severe intellectual disability and was more or less independent but very low in everyday functioning. In 1994, she was described as having severe intellectual disability and needing help for everything. At some time, she was on anti epilepsy medication. In 1996, she was hospitalized in Etanim hospital for intellectual disability and during her stay they found no psychotic signs.

She does not talk; she understands what is going on but pays little attention. She eats independently, needs help dressing, showering, and hygiene. She does not use cash, telephone, and other everyday actions, and she has no friendship or social ability. She co-operates and is helpful. Does not know the time of day, seasons, holidays, the time, reading etc. and is very passive in all activities. She looks happy when music and different activities are going on but does not actively participate. She shows no more stiffness or shaking and is quiet and calm, smiles and turns pages all day.

The psychologist reported: she has no visual signs of dismorphia. At the start she did not co-operate. She had no eye contact or reaction to her name or reaction to what goes on around her and showed no interest in things presented to her. Later in the examination she did react to her name and had some eye contact but spasmodically. She has no affect. She has no interest in content of newspapers but enjoys turning the pages. She laughs a lot and rarely cries. She is all the time accompanied by a staff member when she walks around. (*Compare with now when she can find her way alone to close by familiar places*.)

The MD reported: physical exam pretty much all normal. Her situation is due to premature birth and organic brain damage in early years.

The psychiatrist reported: when she came to Maon Roglit in 1995 it was hard for her to acclimatize and she was very violent, but she has now calmed down. Her medication is better balanced.

In the current diagnosis (2006) that was made by the diagnosis center “Talpiot” Jerusalem – it was found that the patient’s functionality is a moderate to low intellectual disability.

The overall recommendation was that she is stable and no changes were suggested.

The patient had a second evaluation in November 2014, by a social worker, psychologist, MD, psychiatrist with telephone contact with Z (older sister) and presence of the second author, her work place manager.

The social worker reported from her interview, observation, and documentation from examination. She wrote that she looks young for her age but is not aware how she appears. She looks at the wall, has little eye contact, does not talk but makes happy noises and sings to herself. She looks disconnected but seems to be aware of what is happening in the room and every sound gets her attention. She goes twice a year to visit the family. She does not communicate but does react to people she knows. She did not react to what she is asked to do. She eats and drinks by herself; she needs a little help in dressing, puts shoes on by herself, and is independent in bathroom but not at night. She has no meaningful social interaction but does respond to her care workers. She knows her daily routine and how to go around the institute.

She likes looking at books and papers, playing ball, and walking around the institute and outside. She likes music, reacts to it, and sings and hums to herself.

The psychologist used a functional questionnaire, clinical observation, some Wexler WPPSI-3, and different non-formal tests. The patient was at first stressed and uncomfortable, looked around the room, had little eye connection but sometimes looked toward the psychologist and after the second author’s help she calmed down and listened to and reacted to him.

Her verbal understanding is higher than her expressive understanding. She could act on and enjoy simple verbal instructions if motivated to do so. Her understanding is improved when she is shown what to. The second author reported that she does put things into other things in her Waldon Lesson.

She does distinguish between strangers and people she knows and acts accordingly. She shows affect to people she loves by smiling and hugging.

The MD gave a physical examination – nothing new everything normal. She does have some autistic characteristics.

The group report stated: moderate to low intellectual disability – no change from 2006 – and recommended that she should be in the Activities of Daily Living (ADL) group.

## Concluding Remarks

The second author reported: “It was fascinating for me to see the great advancement of the patient following her Waldon Approach lessons over an eighteen month period, and in parallel to see the improvement in her everyday life in the activity of the institute.”

There has been a significant improvement in the patient’s group participation, facial expression, and general demeanor. During the period of this report, she received no other therapy or intervention beyond that provided at Maon Roglit, which itself had not changed during this period. Even though there was no change in her diagnosis of moderate to low intellectual disability between the two assessments in 2006 and 2014 and the patient remains without speech, there has been a real, meaningful, and noticeable change in her life. She joins in activities, spreads sandwiches, and dances at parties. The patient shows more confidence and is more open to hold hands during a dance with another resident. She seems to be calmer and more active in helping the carer and in performing tasks. The patient also has more confidence in going by herself to the Waldon Approach lessons or music lessons – the carer, making sure that she arrives by watching her from far away or calling by phone. After working with the patient for approximately 3 years before she started the Waldon Approach lessons, there is certainly visible progress in general and in specific tasks or situations, from which she appears to derive pleasure.

We believe that this case represents the purest possible demonstration of the efficacy of the Waldon Approach with patients who have intellectual disability with traces of ASD. This patient had no change in circumstances and no different therapies before, during, or after the trial period so that any changes in behavior for better or for worse could be directly attributed to the intervention. The author is participating in a multicenter-controlled trial of the Waldon Approach so this report should be regarded as preliminary findings, which should persuade others to design their own studies on this movement based therapy.

Further information on the Waldon Approach, including several longitudinal case reports, can be found in *Autism and Understanding: The Waldon Approach to Child Development* (1) and http://www.autismandunderstanding.com/. The book was reviewed by Daniel S. Posner in The Journal of Autism and Developmental Disorders (5).

## Limitations of the Study

The limitation of this study is that there are no standard scales of autism and behavior, which could validate the findings. It is a retrospective study written in response to changes in the patient’s behavior, and it was triggered by the changes in her and in other patients during the period of treatment.

## Author Contributions

WGS used the Waldon Approach with the subject of the case report at Maon Roglit. EG is the manager of the group to which the subject belonged at Maon Roglit.

## Conflict of Interest Statement

The first author works as a practitioner of the Waldon Approach and has published a book on the topic (Ref. 1); the second author is in training to become a Waldon practitioner.

## Supplementary Material

For further reading, please find full details in the Supplementary Material available at http://journal.frontiersin.org/article/10.3389/fpubh.2016.00050
