# Supplementary material for: Case Report on the Use of the Waldon Approach on an Adult with Severe to Moderate Intellectual Disability with Autistic Tendencies
Source: Front Public Health. 2016 Mar 29;4:50. doi: 10.3389/fpubh.2016.00050 (PMC4811026; doi:10.3389/fpubh.2016.00050)
Supplement: Supplementary file 1 [file Presentation_1.pdf]

FURTHER READING

Berger J. The Waldon Approach to Educating Developmentally Backward Children: A Feasibility Study within a School Setting (1985). Available from: http://www.autismandunderstanding.com/waldon-approach_03.php

Bornstein M, Hahn CS, Suwalsky JT. Physically-developed and exploratory young infants contribute to their own long-term academic achievement. *Psychol Sci* (2013) **24**(10):1906–17.

Bruner JS. The act of discovery. *Harvard Educ Rev* (1961) **31**:21–32.

Donnellan A, Hill DA, Leary MR. Rethinking autism: implications of sensory and movement differences for understanding and support. *Front Integrative Neurosci* (2013) **6**:124. doi: 10.3389/fnint.2012.00124

Klin A, Jones W, Schultz R, Volkmar F. The enactive mind, or from actions to cognition: lessons from autism. *Philos Trans Royal Soc London Biol Sci* (2003) **358**(1430):345–60.

Lloyd M, MacDonald M, Lord C. Motor skills of toddlers with autism spectrum disorders. *Autism* (2013) **17**:133–46.

Thelen E. Dynamic systems and the complexity of change. *Psychoanal Dialogues* (2005) **15**(2):255–83.

Torres EB, Donnellan AM, editors. *Autism: The Movement Perspective*. Lausanne: Frontiers Media. (2013). doi: 10.3389/978-2-88919-509-1

Trevarthen C, Delafield-Butt JT. Autism as a developmental dis-order in intentional movement and affective engagement. *Front Integr Neurosci* (2013) **7**:49. doi: 10.3389/fnint.2013.00049
